# Pentafluorosulfanyl (SF_5_) as a Superior ^19^F Magnetic Resonance Reporter Group: Signal Detection and Biological Activity of Teriflunomide Derivatives

**DOI:** 10.1021/acssensors.1c01024

**Published:** 2021-10-20

**Authors:** Christian Prinz, Ludger Starke, Tizian-Frank Ramspoth, Janis Kerkering, Vera Martos Riaño, Jérôme Paul, Martin Neuenschwander, Andreas Oder, Silke Radetzki, Siegfried Adelhoefer, Paula Ramos Delgado, Mariya Aravina, Jason M. Millward, Ariane Fillmer, Friedemann Paul, Volker Siffrin, Jens-Peter von Kries, Thoralf Niendorf, Marc Nazaré, Sonia Waiczies

**Affiliations:** † Berlin Ultrahigh Field Facility (B.U.F.F.), Max Delbrück Center for Molecular Medicine in the Helmholtz Association, Robert Rössle Straße 10, 13125 Berlin, Germany; ‡ Experimental and Clinical Research Center, a joint cooperation between the Charité - Universitätsmedizin Berlin and the Max Delbrück Center for Molecular Medicine in the Helmholtz Association, Robert Rössle Straße 10, 13125 Berlin, Germany; § Medicinal Chemistry, Leibniz-Institut für Molekulare Pharmakologie (FMP), Robert Rössle Straße 10, 13125 Berlin, Germany; ∥ Screening Unit, Leibniz-Institut für Molekulare Pharmakologie (FMP), Robert Rössle Straße 10, 13125 Berlin, Germany; ⊥ Physikalisch-Technische Bundesanstalt (PTB), Abbestraße 2-12, 10587 Berlin, Germany; # Charité − Universitätsmedizin Berlin, corporate member of Freie Universität Berlin, Humboldt-Universität zu Berlin, and Berlin Institute of Health (BIH), Charitéplatz 1, 10117 Berlin, Germany

**Keywords:** SF_5_, teriflunomide, DHODH, fluorine, MRI, MRS

## Abstract

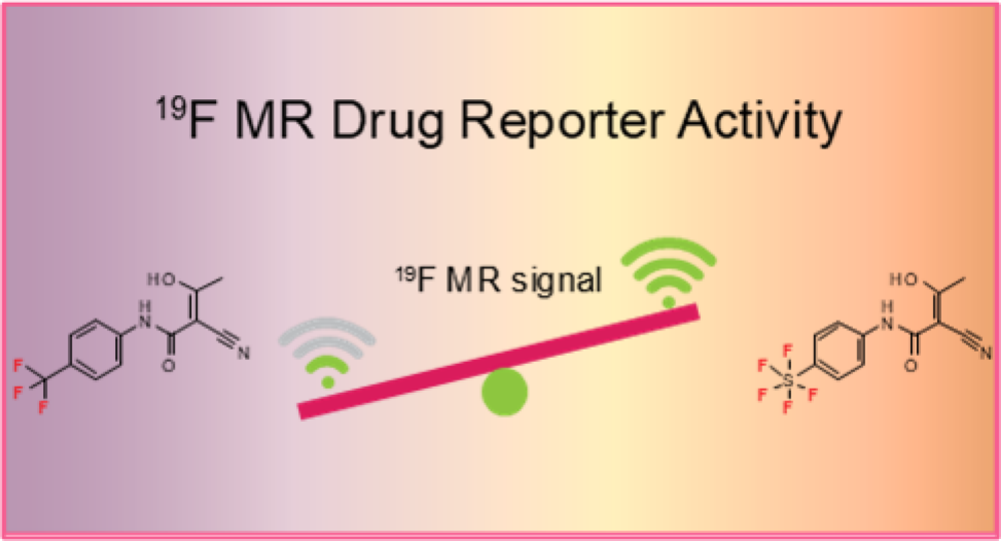

Fluorine (^19^F) magnetic resonance imaging (MRI) is severely limited by a low signal-to noise ratio (SNR), and tapping it for ^19^F drug detection in vivo still poses a significant challenge. However, it bears the potential for label-free theranostic imaging. Recently, we detected the fluorinated dihydroorotate dehydrogenase (DHODH) inhibitor teriflunomide (TF) noninvasively in an animal model of multiple sclerosis (MS) using ^19^F MR spectroscopy (MRS). In the present study, we probed distinct modifications to the CF_3_ group of TF to improve its SNR. This revealed SF_5_ as a superior alternative to the CF_3_ group. The value of the SF_5_ bioisostere as a ^19^F MRI reporter group within a biological or pharmacological context is by far underexplored. Here, we compared the biological and pharmacological activities of different TF derivatives and their ^19^F MR properties (chemical shift and relaxation times). The ^19^F MR SNR efficiency of three MRI methods revealed that SF_5_-substituted TF has the highest ^19^F MR SNR efficiency in combination with an ultrashort echo-time (UTE) MRI method. Chemical modifications did not reduce pharmacological or biological activity as shown in the in vitro dihydroorotate dehydrogenase enzyme and T cell proliferation assays. Instead, SF_5_-substituted TF showed an improved capacity to inhibit T cell proliferation, indicating better anti-inflammatory activity and its suitability as a viable bioisostere in this context. This study proposes SF_5_ as a novel superior ^19^F MR reporter group for the MS drug teriflunomide.

More than one-third of prescribed drugs contain fluorine (^19^F), which generally improves their pharmacokinetic properties.^1−5^ Fluorination also opens an opportunity to noninvasively study drugs in vivo using ^19^F magnetic resonance imaging (MRI). This prospect heralds an age when exact locations and concentrations of drugs can be determined in patients to inform drug therapies.^2−8^ The signal-to-noise ratio (SNR) achieved with ^19^F MRI is limited because of the low availability of ^19^F nuclei in vivo. Additionally, specific ^19^F magnetic resonance (MR) properties (chemical shift, spectral shape, e.g., full width at half maximum or FWHM, spin–lattice (*T*_1_) and spin–spin (*T*_2_) relaxation times) and pharmacological properties (metabolism, protein binding, etc.) could influence SNR and ^19^F MR signal detection.

The application of ^19^F MRI continues to expand, e.g., to track immunotherapies,^9^ investigate temperature-dependent molecular switches,^10^ image tumors using pH-responsive probes,^11^ or monitor inflammatory processes in vivo.^12,13^ The implications of ^19^F MR methods to study physiological, enzymatic, and other metabolic processes in vitro and in vivo have long been recognized.^1,14^ Molecular modifications involving introduction of ^19^F side groups have served several purposes, e.g., as diagnostic biomarkers, where ^19^F tracers or contrast agents are implemented to interrogate function in biological systems.^1^

Fluorinated drugs can be detected in vivo using ^19^F MR spectroscopy (MRS) methods.^15^ Recently, we detected teriflunomide (TF), a dihydroorotate dehydrogenase (DHODH) inhibitor, in an animal model of multiple sclerosis (MS) using nonlocalized ^19^F MRS.^16^ TF inhibits the mitochondrial DHODH enzyme that catalyzes the synthesis of the pyrimidine nucleotide precursor orotate necessary for DNA replication, including CNS-specific T cells that are key players in the MS pathology, thereby exerting its anti-inflammatory action in MS.^17^ Despite recent advances to improve radiofrequency coil sensitivities,^18^ there are still limitations that impede detection of ^19^F drugs in vivo with ^19^F MRI or localized ^19^F MRS, while these methods will be essential to locate drugs within specific tissue and study their distribution in vivo. SNR was a major limitation in our previous study due to the low TF availability in vivo, thereby prohibiting imaging or localized MRS to specify its location in vivo.^16^

The pentafluorosulfanyl (SF_5_) group has gained an immanent role in organic materials, polymers, and liquid crystals and has also recently emerged as a bioisostere for lipophilic groups like CF_3_, *tert*-butyl, halogen, or nitro groups in biologically active compounds.^19^ SF_6_ has been studied extensively in the gas phase as a diagnostic tool for pulmonary ^19^F MRI.^20−25^ However, surprisingly, the corresponding derived organic -SF_5_ group has thus far not been investigated as a ^19^F MRI reporter group.

In this study, we investigated different modifications to the CF_3_ group of TF. This included the addition of two symmetrical CF_3_ groups to increase the number of ^19^F atoms and the introduction of a SF_5_ group and a trifluoromethoxy-group to probe the effects of altering the chemical environment of ^19^F atoms while leaving their number constant, to improve their SNR and promote drug monitoring in vivo. In parallel, we also aimed to preserve or even improve the pharmacological activity of TF. Generally, the introduction of fluorine substituents enhances the potency of DHODH inhibition of TF.^26^ Here, the CF_3_ group plays an important role in stabilizing the bioactive conformation of TF.^27^ The SF_5_ group can be considered a bioisostere of CF_3_-groups in drug compounds.^28^ After a thorough characterization of the pharmacological properties and ^19^F MR properties of the synthesized derivatives, we selected and optimized suitable MR sequences, compared their SNR efficiency in imaging the derivatives, and aligned this with their capacity to inhibit T cell proliferation. Ultimately, we selected the best suited candidate for an ex vivo demonstration of ^19^F MRI in the murine stomach.

## Experimental Section

### Synthesis of TF and Its Derivatives

**Figure 1:**
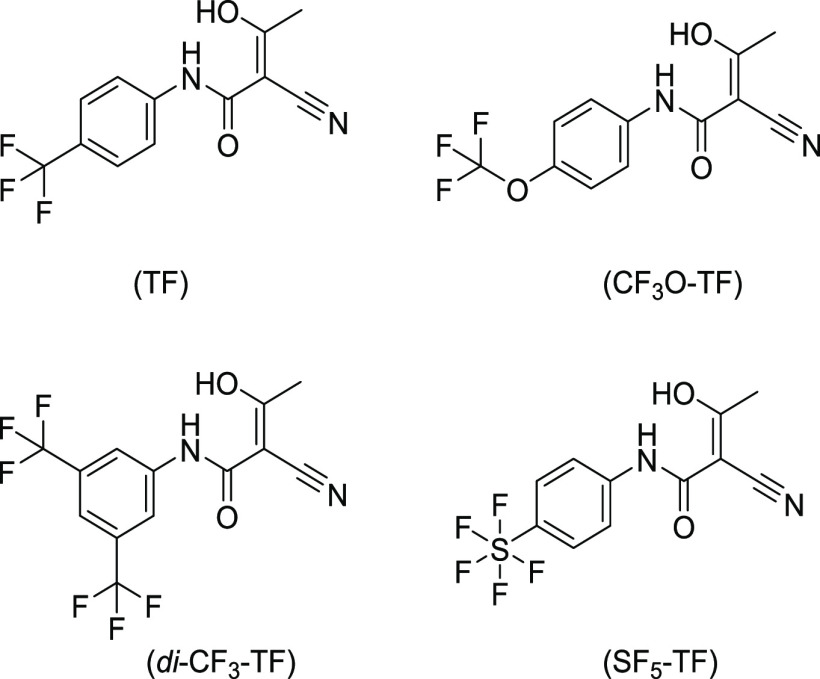
Chemical structures of the synthesized teriflunomide derivatives.

Fluorinated anilines were coupled with isoxazol acid chloride by a Schotten–Baumann reaction,^29,30^ and the isoxazol ring of the resulting leflunomide prodrug structure was hydrolyzed for conversion into the teriflunomide derivatives^31^ (Supporting Information: Chemistry, Figure S1). Derivatives of TF (Figure 1) resulted from a substitution of the *para* CF_3_-group on the benzene ring with a trifluoromethoxy CF_3_O-R group, two chemically symmetrical *meta* CF_3_ groups, and one *para* pentafluorosulfanyl SF_5_ group.

### Pharmacological Activity

The inhibitory activity of TF derivatives on the DHODH enzyme was measured with a colorimetric enzyme activity assay. Drug concentration ranged from 50 μM serially diluted in DMSO to 4.9 nM with a final DMSO concentration of 1%. DHODH inhibition was studied by monitoring the reduction of 2,6-dichloroindophenol (DCIP) (Alfa Aesar) via color change (blue to colorless, loss of absorbance at 620 nm). The colorimetric reaction is associated with the oxidation of l-dihydroorotate (l-DHO) (Alfa Aesar) that is catalyzed by the DHODH enzyme.^32−35^ The reaction was performed in duplicate in volumes of 30 μL in a 384-well plate in 50 mM tris–HCl buffer, containing 73 nM coenzyme Q10 (Selleckchem), 0.1% Triton X-100 (Sigma-Aldrich), 150 mM KCl (Sigma-Aldrich), 211 nM DCIP (Thermo Fisher), and 7.5 nM DHODH (Biozol). The reaction was started by the addition of the substrate l-DHO, performed at 30 °C, and monitored over 60 min using a Tecan Safire2 Multimode Microplate Reader (Tecan).

### Biological Activity

The inhibitory activity of TF derivatives on T cell proliferation was studied after isolating peripheral blood mononuclear cells (PBMCs) from healthy volunteers. Informed, signed consent was obtained for collecting blood samples. Approval was granted from the ethics commission of the Charité (EA1/023/15). Compounds were dissolved in DMSO (19 mM) and further diluted to final concentrations ranging from 0.39 to 50 μM.^36^ Compounds (10 μL) were plated on 96-well plates and stored at 4 °C.

Blood (in EDTA) was withdrawn from two male healthy volunteers (aged 25–35), diluted 1:2 in PBS, and layered on Lymphopure (BioLegend) for separating cell populations using the principle of Ficoll density gradient centrifugation (764 g, 40 min, RT, no brake). PBMCs were cautiously collected from the corresponding phase and diluted in PBS. After two washing steps in PBS, the cell pellet was resuspended in a HEPES–RPMI 1640 buffer and counted using a Neubauer cell-counting chamber.

Isolated PBMCs were labeled with carboxyfluoresceinsuccinimidylester (CFSE) (BioLegend) as described elsewhere.^37^ The fluorescent dye CFSE is taken up into the cells. Dilutions of CFSE in daughter cells (1:2 as a result of cell division) result in sequential losses in fluorescence intensities. Labeling was performed according to the manufacturer’s descriptions using 5 μM CFSE, and staining was quenched using a medium containing 10% FCS. Cells were recounted and resuspended in a cell culture medium (5% human AB serum, 1% GlutaMax (×100, Gibco), 10 mM HEPES (Gibco), and 1% penicillin (10 U/μL)/streptomycin (10 μg/μL) (Gibco) in an RPMI 1640 medium (Gibco)) at a concentration of 10^6^ cells/mL. To induce T cell proliferation, isolated PBMCs were incubated with the superantigen-similar polyclonal stimulating reagent CytoStim (Miltenyi Biotec) following the manufacturer’s instructions. CytoStim was added to the cell suspensions (0.2%).

Cells with or without CytoStim were distributed in 96-well plates (in triplicate, with final volumes of 200 μL per well) and kept at 37 °C and 5% CO_2_ for 72 h. Wells without DHODH inhibitors (TF or its derivates) served as positive proliferation controls, and wells without T cell stimuli and inhibitors served as negative controls. After incubation, cells were centrifuged, washed with PBS, and resuspended in FACS buffer (0.5% BSA and 0.1% NaN_3_ (Sigma-Aldrich) in PBS). Propidium iodide (PI) (BioLegend) was added to each well approximately 5 min before the measurement to stain the dead cell population. The fluorescence of CFSE labeling and PI staining was analyzed by flow cytometry using a BD LSRFortessa flow cytometer (BD Biosciences).

### MR Methods

MR experiments were performed on a 9.4 T MR scanner (Bruker Biospec, Ettlingen, Germany). All DHODH inhibitors (TF or its derivates) were diluted in either 100% DMSO (Roth) or 100% human serum. The human serum was prepared from whole blood withdrawn from a male healthy volunteer (aged 25) and allowed to stand for 15–30 min at room temperature; following centrifugation (21.1 g, 10 min, RT), the supernatant (serum) was transferred into a new container. Serum samples were stored at −20 °C. Phantoms were prepared in 2 and 1 mL syringes to characterize the spectra, chemical shifts, relaxation times, and signal-to-noise (SNR) efficiencies. Teriflunomide (Sigma-Aldrich and Genzyme) was used as a reference compound. Concentrations of the compounds were adjusted as indicated in Table S1.

For phantom experiments, a home-built dual-tunable ^19^F/^1^H mouse head RF coil was used.^12^ MR measurements were performed at room temperature (RT).

A global single pulse spectroscopy (TR = 1000 ms, TA = 8 s, nominal flip angle (FA) = 90°, block pulse, 4096 points, dwell time = 0.02 ms, excitation pulse = 10,000 Hz, spectral bandwidth = 25,000 Hz) was used to detect the ^19^F signal and to make frequency adjustments. Chemical shifts are referenced to trichlorofluoromethane, CFCl_3_ (δ_F_ = 0 ppm).

For determining *T*_1_ in low concentrated serum samples, global spectroscopy with different TRs (TR = 50–8000 ms) was used. For spectroscopically determining *T*_2_, a CPMG sequence was used (TR = 5000 ms, 25 echoes, echo spacing = 2.8 ms, excitation pulse = 5000 Hz, spectral bandwidth = 25,000 Hz).

*T*_1_ mapping was performed using RARE (rapid acquisition with relaxation enhancement): TE = 4.6 ms, echo train length (ETL) = 4, FOV = [16 × 16] mm^2^, matrix size = 64 × 64, with 9 variable repetitions times (TR = 25–8000 ms). *T*_2_ mapping was performed using a multislice multiecho sequence: TR = 2000 ms, FOV = [16 × 16] mm^2^, matrix size = 64 × 64, with 25 different TEs (TE = 40–1000 ms in steps of 40 ms for long *T*_2_ and TE = 8–200 ms in steps of 8 ms for short *T*_2_).

To determine the most SNR efficient MR technique for acquiring ^19^F MRI of TF and its derivatives, we optimized the parameters (Table S3) of three MR sequences: RARE, bSSFP (balanced steady-state free precession), and UTE (ultrashort echo time). For all methods, the image geometry was set to FOV = [28 × 28] mm^2^, matrix size = 96 × 96 in DMSO, matrix size = 64 × 64 in serum, and slice thickness = 5 mm. Pulse sequence parameters were optimized based on the relaxation times of each compound in DMSO and serum.^38,39^

For RARE, the receiver bandwidth was set to 10 kHz to maximize SNR but limit chemical shift artifacts. Centric encoding was used, and the echo spacing was kept minimal for both long *T*_2_ conditions, i.e., in DMSO (TE_DMSO_ = 12.12 ms), and short *T*_2_ conditions, i.e., in serum (TE_serum_ = 2.29 ms). For the latter condition, a RARE sequence with gradient pulses optimized for short echo spacing (RAREst) was used. We used a flip-back module and, based on simulations, chose the highest possible ETL with a full width at half maximum (FWHM) of the point spread function (PSF) below 1.5 pixels. Given these parameters, we optimized the repetition time (TR) based on the steady state signal intensity equation.^38^

For bSSFP, the receiver bandwidth was set to 100 kHz to achieve a short TR = 2.6 ms (TE = 1.3 ms) and thus stability regarding banding artifacts. For each compound and condition, the flip angle α_bSSFP_ was adjusted to
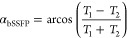


For UTE, we used an FID readout (TE = 0.27 ms) and a read bandwidth of 20 kHz. We used TR = 100 ms and the calculated Ernst angle α_E_ as the flip angle
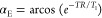


Animal experiments were conducted in accordance with the procedures approved by the Animal Welfare Department of the State Office for Health and Social Affairs Berlin (LAGeSo) and conformed to guidelines to minimize discomfort to animals (86/609/EEC).

The same molar concentrations of TF (12.15 mg/mL) and SF_5_-TF (10 mg/mL) suspended in 0.6% carboxymethyl cellulose were administered orally (bolus volume of 800 μL) to an anesthetized (intraperitoneal injection, 7.5 mg/kg of xylazine and 100 mg/kg of ketamine in NaCl) C57BL/6N mouse. After 20 min, the animal was sacrificed by an overdose of the anesthetic, the esophagus and duodenum were closed with a surgical suture, and the stomach was removed. An ex vivo phantom was prepared in a 5 mL tube filled with 4% paraformaldehyde (Santa Cruz Biotechnology). We used RARE for anatomical ^1^H MRI of the stomach: TR/TE = 2000 ms/10 ms; TA = 1 min, 4 s; FOV = [28 × 28] mm^2^, and matrix = 256 × 256, slice thickness = 0.5 mm, and an optimized UTE sequence for ^19^F MRI of TF and SF_5_-TF: TR/TE = 100 ms/0.27 ms; TA = 2 h, 30 min, slice thickness = 5 mm, FA_TF_ = 25°, FA_SF5-TF_ = 42°, FOV = [28 × 28] mm^2^, and matrix = 32 × 32.

### Data Analysis

For DHODH activity, time–absorbance curves reflect the enzymatic activity over time. We calculated the initial slope of these curves to quantify the initial velocity of the enzyme reaction. A *Z*′ factor was calculated^40^ using an in-house online *Z*′ calculator to assess the quality of the screening method (http://www.screeningunit-fmp.net/tools/z-prime.php). Screening assays with *Z*′ values between 0.5 and 1 are categorized as excellent.^40^

For T cell proliferation, we analyzed flow cytometry data using FlowJo (software version 10.5.3, FlowJo LLC, Figure S2). The lymphocyte population was selected from the forward (FSC-A)/sideward scatter (SSC-A) and gated for single cells (FSC-A/FSC-H). Dead cells were excluded by gating for the cell population SSC-A/PI without PI staining. From this population, the CFSE-labeled cells were gated and displayed in a histogram, dividing the different generations of cells from cell divisions. The percentage of proliferating cells in relation to the originally labeled parental cell population was calculated.

A *Z*′ factor (see the DHODH assay) was calculated from the number of proliferating cells in unstimulated negative and stimulated untreated positive controls. Additionally, the stimulation index (SI) was determined from the proportion of proliferating cells from nonproliferating ones
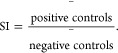


Data from triplicates from each donor were combined so that each concentration point is represented by three measurement points.

MR image processing and spectral analysis and processing were performed in MATLAB R2018a (MathWorks, Inc.). MR data was analyzed as detailed in our previous work.^41^ Briefly, we quantified the spectral signal intensity, performed a Fourier transform to obtain magnitude spectra, and determined the chemical shift. We estimated the *T*_1_ and *T*_2_ time constants by fitting the measured signal intensities in MR magnitude images to the equations for *T*_1_ and *T*_2_ relaxations. SNR estimations for RARE, bSSFP, and UTE measurements and image preparation of ex vivo samples were performed as previously described.^42^ ImageJ^43^ was used for further image analysis. We calculated the SNR efficiency (SNR/sqrt(time)) per mol to determine the compounds beneficial for in vivo application.

## Results and Discussion

### Inhibitory Activity of TF Derivatives In Vitro

The trifluoromethoxy-substituted TF (CF_3_O-TF) and pentafluorosulfanyl-substituted TF (SF_5_-TF) demonstrated equal or even better pharmacological and antiproliferative activities compared to TF (CF_3_-TF) as shown by the IC_50_ values for DHODH inhibition in in vitro enzyme and proliferation assays (Table 1). We calculated the IC_50_ values for pharmacological DHODH inhibitory activity from dose–response curves and by fitting a dose–response model to the data points (Figure S3). The impact of TF and its derivates to inhibit T cell proliferation was also determined from the IC_50_ values from dose–response curves and by fitting a dose–response model to the data points (Figure S3). The T cell stimulation index yielded 47.3.

**Table 1:** IC_50_ Values and Confidence Intervals of the Enzyme Inhibition and Cell Proliferation Assays^a^

	DHODH inhibition			cell proliferation inhibition		
compound	IC_50_ [μM]	lower limit CI [μM]	upper limit CI [μM]	IC_50_ [μM]	lower limit CI [μM]	upper limit CI [μM]
TF	0.54	0.32	0.77	24.25	19.01	29.52
CF_3_O-TF	0.33	0.11	0.55	10.98	8.65	13.31
*di*-CF_3_-TF	1.32	0.53	2.11	39.73	6.88	72.60
SF_5_-TF	0.58	0.42	0.73	8.48	8.04	8.92

a The *Z*′ value for the proliferation assay was 0.893, which is comparable to the *Z*’ value obtained in the enzyme inhibition assay. A *Z*′ value of >0.5 confirms the robustness of the assay. Therefore, unstimulated cells could be safely discriminated from stimulated controls.

For TF and its derivatives, we calculated the IC_50_ values and 95% confidence intervals, for both DHODH and T cell proliferation assays (Figure S3). The activity of CF_3_O-TF to inhibit DHODH was marginally higher than that of TF as shown by the lower IC_50_, while that of SF_5_-TF was similar, and that of the difluoromethyl-substituted *di*-CF_3_-TF was lower (Figure S3 and Table 1). In T cell proliferation experiments, both CF_3_O-TF and SF_5_-TF showed a considerably greater inhibitory activity than TF, as shown by the dose–response curves (Figure S3B) and IC_50_ values (Table 1). Compared to TF, SF_5_-TF revealed the best inhibitory activity (IC_50_ 8.48 μM).

Substitution of the CF_3_ group with a SF_5_ group is expected to result in changes in the side chain geometry and electron density. The SF_5_ group is characterized by a higher electronegativity and lipophilicity.^19^ The increased T cell inhibitory activity by SF_5_-TF suggests an increased cellular uptake in T cells as a result of more efficient permeation through cell membranes due to increased lipophilicity, as has been shown in trypanothione reductase inhibitors exhibiting increased cellular activity and membrane permeability following introduction of SF_5._^44^

We also performed a cell growth inhibition and cytotoxicity test in HepG2 cells (Supporting Information: Toxicology). All compounds showed a similar impact on cancer cell growth and cytotoxicity to that of TF (Figure S4). While T cell inhibitory activity of SF_5_-TF was enhanced, its impact on cytotoxicity was similar to that of TF. Furthermore, nucleic acid staining using propidium iodide (PI) did not reveal cytotoxic effects in T cells, even at higher concentrations (Figure S5).

### ^19^F MR Relaxation and Chemical Shifts of TF Derivatives

**Figure 2:**
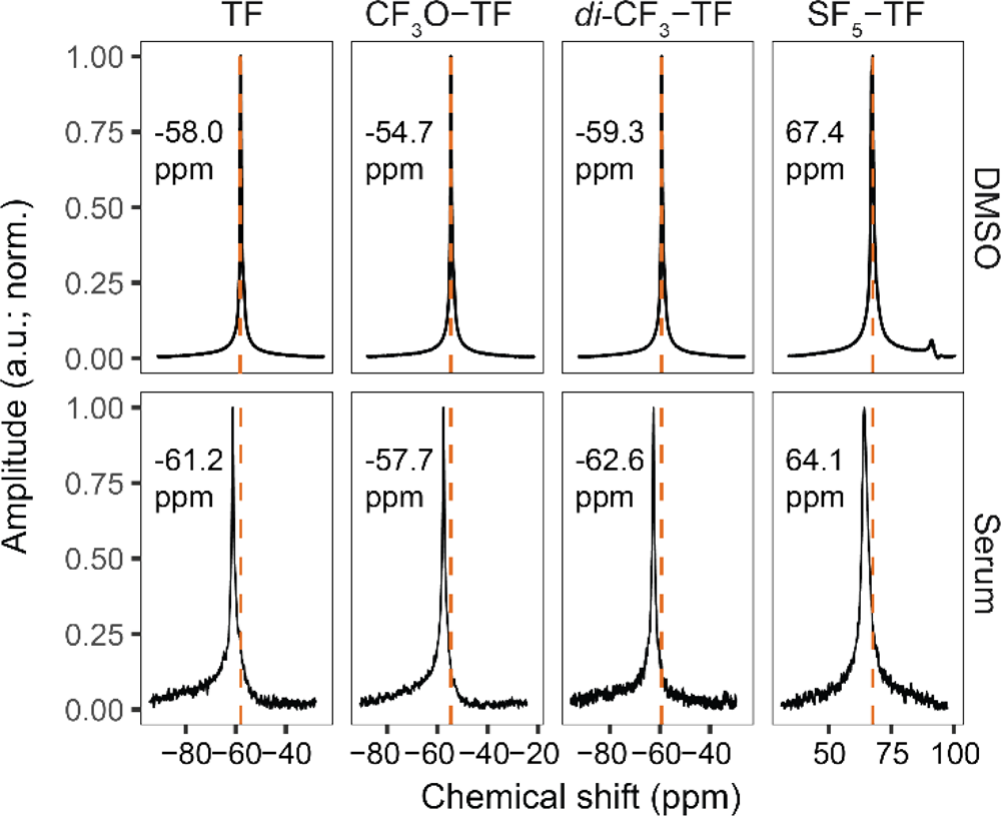
^19^F MR spectra of teriflunomide and its derivatives. Signal acquired (normalized amplitudes) from compounds dissolved in DMSO (upper panels) or human serum (lower panels) obtained by a global single-pulse spectroscopy (TR = 1000 ms and TA = 8000 ms).

We studied the ^19^F MR properties of all TF derivatives in DMSO and in human serum. We used serum to simulate the in vivo situation. When TF is administered to patients, it becomes strongly protein bound (e.g., in blood, it is >99% bound to protein^45^). Since the solubility of TF compounds in serum was lower than in DMSO, lower concentrations of TF compounds were available in serum (Table S1); this resulted in an expected lower SNR (Figure S6). TF, CF_3_O-TF, and *di*-CF_3_-TF exhibit a single peak at −58, −55, and −59 ppm, respectively, in DMSO (Figure 2). SF_5_-TF shows a main peak at 67.4 ppm and a smaller peak at 91.7 ppm at a ratio of 4:1 (Figure 2 and Figure S7). Due to the geometric orientation of the five fluorine atoms to the phenyl moiety, one positioned axially and four in equatorial positions, the main peak shows a line splitting (doublet) and the smaller peak shows an apparent 1:4:6:4:1 intensity pentet (Figure S7).^46^

Spectra of the compounds solubilized in serum were similar to those in DMSO; although we observed a slight change in the chemical shift and peak broadening (Figure 2, lower panels), also quantified from the full width at half maximum (FWHM) of the peaks (Table S2).

Interestingly, increasing the number of fluorine atoms on a drug structure is not the only factor that affects the effective SNR. Our results indicate that addition of the SF_5_-group to the molecular structure reduces both the transversal relaxation time *T*_2_ and longitudinal relaxation time *T*_1_.

Spin–lattice relaxation times (*T*_1_) were measured for all TF derivatives (Table 2). Compared to TF in DMSO, *di*-CF_3_-TF had an almost equal *T_1_* relaxation time, CF_3_O-TF had a prolonged *T_1_* relaxation time, and SF_5_-TF had a substantially reduced *T_1_* relaxation time. In serum, *T*_1_ was equal for TF but was reduced for CF_3_O-TF, *di*-CF_3_-TF, and SF_5_-TF, compared to the same compounds in DMSO.

**Table 2:** Comparison of *T*_1_ and *T*_2_ Relaxation Times in DMSO and Serum of TF and Its Derivatives to Optimize the SNR Efficiency of the ^19^F MR Acquisition Method

	*T*_1_ (ms)		*T*_2_ (ms)	
compound	DMSO	serum	DMSO	serum
TF	1003	1017	508	5
CF_3_O-TF	1666	753	942	8
*di*-CF_3_-TF	1098	875	642	5
SF_5_-TF	371	331	68	6

Spin–spin relaxation times (*T*_2_) were also measured for all compounds (Table 2). Compared to TF in DMSO, CF_3_O-TF and *di*-CF_3_-TF had longer spin–spin relaxations, while SF_5_-TF had a markedly shorter spin–spin relaxation. The *T*_2_ in serum also dramatically dropped to values between 5 and 8 ms.

While a reduction in *T*_2_ requires MR methods with faster signal acquisition (e.g., UTE), a reduction in *T*_1_ reduces the time required between different excitations, thereby enabling more efficient data acquisition. The reductions in relaxation times could be attributed to dipole–dipole interactions and chemical shift anisotropy.^47,48^ Besides modifications of side chains, the addition of a paramagnetic dopant such as lanthanide chelates, in particular, gadolinium-containing contrast agents, has been used for shortening the *T*_1_*.*(49) For ^19^F MR applications, ^19^F nanoparticles were functionalized with Gd(III) complexes for modulating the ^19^F signal.^50^ Increased ^19^F MR signals from ^19^F nanoparticles were observed in close proximity to blood–brain barrier disruptions, indicating a *T*_1_ shortening effect not only for ^1^H but also for^19^F as a result of the paramagnetic contrast agent Gd–DTPA leakage.^51^

### Impact of Serum on ^19^F MR Relaxation

Differences in chemical shifts and relaxation times can be attributed to the different physicochemical properties of the ^19^F groups. These properties were also severely affected by the serum environment. SF_5_-TF is administered as a suspension, and SF_6_ is administered as a gas. All six fluorines on SF_6_ produce one signal, whereas the SF_5_ group has an AX4 spin system and produces two separate signals. Most notably, the ^19^F *T*_2_ relaxation times of TF and its derivatives were drastically shortened in serum. The increase in FWHM of the ^19^F MR spectra in the serum compared to DMSO also suggests a *T*_2_* shortening.^52^ The observed shortening in relaxation is presumably attributed to unspecific binding of the compounds to serum proteins.^53^ Similar to SF_5_-substituted TF, the fluorinated gas SF_6_ exhibits a short transversal relaxation time.^20^ SF_5_ is an organic derivative of SF_6_ in which one of the fluorine atoms is replaced by an organic residue. However, there are notable *T*_1_ and *T*_2_ differences between SF_5_-TF and SF_6_.^20,21^ At 9.4 T, the *T*_1_ of SF_6_ at room temperature (300 K) was reported to be <200 ms^21^ and is even shorter (1.24 ms at 1.9 T) when administered to rats as a gas (80%, 630 Torr barometric pressure).^20^ The *T*_1_ of SF_5_-TF was not strongly reduced in the presence of serum (Table 2), and we would expect similar values for this compound in vivo. On the other hand, the *T*_2_ of SF_5_-TF was strongly reduced (by 90%) in the presence of serum. Differences between SF_5_-TF and SF_6_ with respect to *T*_1_ and *T*_2_ can be attributed to differences in chemical environments.^23^

Recently, we could detect TF in vivo when employing ^19^F MRS.^16^ The above limitations in relaxation times, particularly when protein bound, restrict SNR. Due to the associated rapid loss in ^19^F signal during *T*_2_ relaxation, signal detection with ^19^F MRI is expected to be more challenging.

### Optimization of ^19^F MR Parameters

We used the determined ^19^F relaxation times for TF and its derivatives to calculate the most efficient parameters for three ^19^F MRI acquisition methods: rapid acquisition with relaxation enhancement (RARE), balanced steady-state free precession (SSFP), and ultrashort echo time (UTE) (Table S3). Selection of the appropriate MR acquisition method according to the MR properties of the compound within a specific environment is essential to acquire data with the best SNR efficiency. A thorough characterization of ^19^F compounds^41^ is typically necessary to adapt SNR efficient ^19^F MR acquisition methods to the specific MR characteristics of these compounds.^38^

We acquired ^19^F MR images of all compounds using the corresponding optimized sequences (Figure S6) and compared SNR efficiencies (SNR per molecule divided by the square root of the acquisition time) to identify the most suited method for each compound, in DMSO and in serum (Table 3). In DMSO, RARE yielded the best SNR efficiency for all compounds, followed by bSSFP and UTE. In DMSO, *di*-CF_3_-TF showed the best SNR efficiency performance compared to TF. In serum, UTE was the MR acquisition method that provided the best SNR efficiency for all TF compounds, particularly for SF_5_-TF. The SF_5_-substituted derivative showed a gain in ^19^F SNR efficiency of ∼3 compared to TF when using the UTE ^19^F MRI method. This translates into a 9-fold reduction in measurement time, which is the primary obstacle of ^19^F MRI for both clinical and preclinical applications. In addition, the SF_5_-TF also showed an increase of ∼3 in inhibiting T cell proliferation.

**Table 3:** SNR Comparison Using Optimized RARE, bSSFP, and UTE Protocols Studying the SNR Efficiency of Compounds (Using SNR per Molecule) in DMSO and in Serum (in % Normalized to TF RARE)

	RARE		bSSFP		UTE	
compound	DMSO	serum	DMSO	serum	DMSO	serum
TF	100	100	63	9	17	163
CF_3_O-TF	106	101	55	20	14	148
*di*-CF_3_-TF	200	162	130	34	33	240
SF_5_-TF	68	76	74	26	32	363

One interpretation of these results is that the increased activity of SF_5_-TF could allow a lower dose to be used while maintaining the same level of therapeutic efficacy. However, a randomized clinical study following 12–14 years of TF treatment showed that a larger proportion of MS patients (51.5%) were relapse-free when receiving 14 mg of teriflunomide, compared to those receiving 7 mg of TF (39.5%).^54,55^ Thus, the higher efficacy of SF_5_-TF might have downstream benefits for MS patients, and future in vivo experiments in the animal model will be needed to clarify whether SF_5_-TF administered at the same dose as TF will be more efficacious at treating neurological diseases. Apart from a ∼3× gain in the ^19^F SNR efficiency in vitro, we observed an SNR gain of ∼2 for SF_5_-TF compared to TF in the stomach ex vivo. This variation in the SNR gain could reflect variations in pH in the stomach that could affect relaxation times of TF.^16^ Future comparative studies are needed to focus on organ systems more relevant to the pathophysiology of MS such as the CNS.

### Biological and ^19^F MR Reporter Activities of TF Compounds

**Figure 3:**
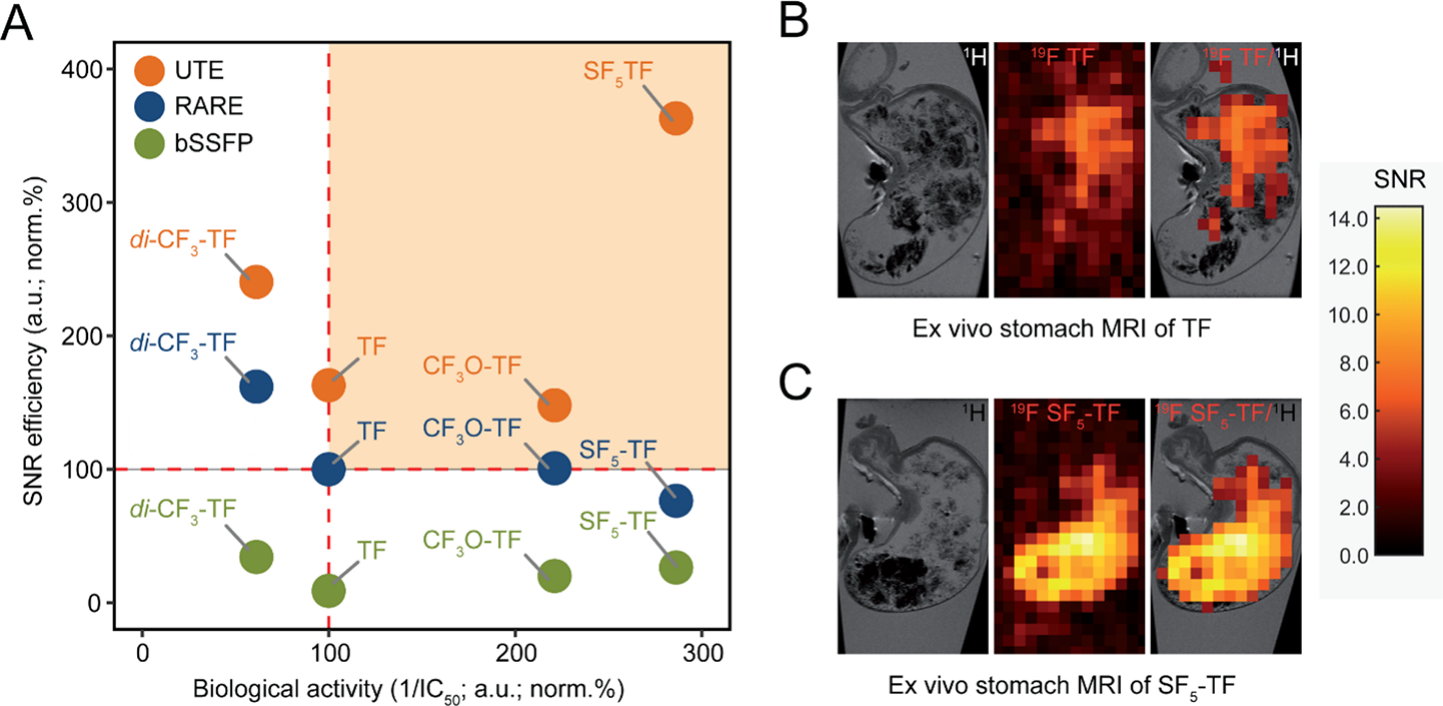
Biological and ^19^F MR reporter activities of TF compounds. (A) Selecting the best ^19^F TF derivative and corresponding MR acquisition method. Drug compounds with the best inhibitory capacity (inverse IC_50_ normalized to TF in percentage) and best SNR efficiency in serum (normalized to TF in percentage using a RARE sequence) obtained using RARE, bSSFP, and UTE sequences are shown in the upper right quadrant (orange region). (B)^19^F MR reporter activity of TF in the stomach of a C57BL/6 mouse ex vivo using an optimized ^19^F UTE MR sequence. Left panel: ^1^H anatomical image of the stomach, middle panel: ^19^F MRI of TF in the stomach, and right panel: ^19^F/^1^H overlay. ^1^H: RARE (TR/TE = 2000 ms/10 ms, TA = 1 min, 4 s) and ^19^F: UTE (TR/TE = 100 ms/0.27 ms, TA = 2 h, 30 min, FA = 25 °). (C) ^19^F MR reporter activity of SF_5_-TF in the stomach of a C57BL/6 mouse ex vivo using an optimized ^19^F UTE MR sequence. Left panel: ^1^H anatomical image of the stomach*,* middle panel: ^19^F MRI of SF_5_-TF in the stomach, and right panel: ^19^F/^1^H overlay. ^1^H: RARE (TR/TE = 2000 ms/10 ms, TA = 1 min, 4 s) and ^19^F: UTE (TR/TE = 100 ms/0.27 ms, TA = 2 h, 30 min, FA = 42 °). SNR is indicated by the color bars.

We aligned the biological activity with SNR efficiency of all ^19^F MRI acquisition methods when comparing TF with its substituted derivatives (Figure 3A). Compared to all compounds, SF_5_-TF showed a clear enhancement in the T cell proliferation inhibitory activity, as well as SNR efficiency under serum conditions when using a UTE sequence. We administered TF and SF_5_-TF orally to C57BL/6 mice using the same molar concentrations (TF =12.15 mg/mL (*n* = 3) and SF_5_-TF = 10 mg/mL (*n* = 3)) and acquired ^19^F MR images of the stomachs ex vivo using the optimized parameters (Figure 3B,C). We observed a clear distribution of both TF and SF_5_-TF in the stomach and measured peak SNR values of 13.2 ± 1.2 (mean ± maximum absolute deviation) for SF_5_-TF in the stomach, compared to 5.8 ± 2.9 for TF (MR Characterization, Figure S8 and Table S4).

## Conclusions

In this study, we introduced modifications, including those of SF_5_, to a pharmacologically active compound, to increase detection in vivo. To our knowledge, this is the first study that has performed an in-depth investigation of ^19^F modifications on pharmacological and biological activities, alongside the MR reporter function. Here, we addressed SNR by modifying the CF_3_ side chain of TF to vary the ^19^F MR properties and identify potential SNR-boosting derivatives. Substituting the CF_3_ side chain by CF_3_O, SF_5_, or *di*-CF_3_ did not compromise the pharmacological and antiproliferative activities. Of note, the superiority of the SF_5_-substituted derivative to inhibit T cell proliferation compared to TF indicates that some fluorination strategies might even improve the biological function.

Our study indicates SF_5_ as a potential theranostic marker for detecting and studying the biodistribution of fluorinated drugs noninvasively, particularly during pathology. However, in vivo studies will now be necessary to study the versatility of the SF_5_ bioisostere in combination with the UTE method for in vivo ^19^F MRI and to determine its superiority over teriflunomide to treat disease in the animal model of multiple sclerosis.

## Supporting Information Available

Supplemental methods and results (chemistry, flow cytometry, enzyme and T cell growth inhibition, toxicology, and MR characterization) (PDF)

## Abbreviations

^19^F: fluorine-19
1H: hydrogen-1/proton
bSSFP: balanced steady-state free precession
CF3: trifluoromethyl
CFSE: carboxyfluoresceinsuccinimidylester
DCIP: dichloroindophenol
DHODH: dihydroorotate dehydrogenase
DMSO: dimethylsulfoxide
FA: flip angle
FID: free induction decay
FOV: field of view
FWHM: full width at half maximum
IC_50_: inhibitory concentration (50%)
l-DHO: l-dihydroorotate
MR: magnetic resonance
MRI: magnetic resonance imaging
MS: multiple sclerosis
PBMCs: peripheral blood mononuclear cells
PSF: point-spread-function
RARE: rapid acquisition with relaxation enhancement
RF: radiofrequency
RT: room temperature
SF5: pentafluorosulfanyl
SNR: signal-to-noise ratio
TA: acquisition time
TE: echo time
TF: teriflunomide
TR: repetition time
UTE: ultrashort echo time
