# Supplementary material for: Pentafluorosulfanyl (SF5) as a Superior 19F Magnetic Resonance Reporter Group: Signal Detection and Biological Activity of Teriflunomide Derivatives
Source: ACS Sens. 2021 Oct 20;6(11):3948–56. doi: 10.1021/acssensors.1c01024 (PMC8630787; doi:10.1021/acssensors.1c01024)
Supplement: Supplementary file 1 — se1c01024_si_001.pdf [file se1c01024_si_001.pdf]

Supplement

Pentafluorosulfanyl (SF_5) as a Superior ^{19}F Magnetic Resonance Reporter Group: Signal Detection and Biological Activity of Teriflunomide Derivatives

Christian Prinz,[1,2] Ludger Starke,[1] Tizian-Frank Ramspoth,[3] Janis Kerkering,[2] Vera Martos Riaño,[3] Jérôme Paul,[3] Martin Neuenschwander,[4] Andreas Oder,[4] Silke Radetzki,[4] Siegfried Adelhoefer,[1] Paula Ramos Delgado,[1,2] Mariya Aravina,[1] Jason M. Millward,[1,2] Ariane Fillmer,[5] Friedemann Paul,[2,6] Volker Siffrin,[2] Jens-Peter von Kries,[4] Thoralf Niendorf,[1,2] Marc Nazaré,[3] Sonia Waiczies *[1,2]

sonia.waiczies@mdc-berlin.de

[1] Berlin Ultrahigh Field Facility (B.U.F.F.), Max Delbrück Center for Molecular Medicine in the Helmholtz Association, Robert Rössle Straße 10, 13125 Berlin, Germany

[2] Experimental and Clinical Research Center, a joint cooperation between the Charité - Universitätsmedizin Berlin and the Max Delbrück Center for Molecular Medicine in the Helmholtz Association, Robert Rössle Straße 10, 13125 Berlin, Germany

[3] Medicinal Chemistry, Leibniz-Institut für Molekulare Pharmakologie (FMP), Robert Rössle Straße 10, 13125 Berlin, Germany

[4] Screening Unit, Leibniz-Institut für Molekulare Pharmakologie (FMP), Robert Rössle Straße 10, 13125 Berlin, Germany

[5] Physikalisch-Technische Bundesanstalt (PTB), Abbestraße 2-12, 10587 Berlin, Germany

[6] Charité – Universitätsmedizin Berlin, corporate member of Freie Universität Berlin, Humboldt-Universität zu Berlin, and Berlin Institute of Health (BIH), Charitéplatz 1, 10117Berlin, Germany

Chemistry

General information

All chemicals were purchased from commercial suppliers: Activate Scientific, Sigma-Aldrich, TCI, Thermo Fisher Scientific, and Alfa Aesar and used as received unless otherwise specified. For all reactions, analytical grade solvents were used. All moisture- sensitive reactions were carried out in oven-dried glassware (135°C). Microwave reactions were performed in a Microwave Biotage Initiator+ with a Robot Sixty autosampler. For structural elucidation ^{1}H-, ^{13}C-, ^{13}C apt- ,^{19}F-, HMQC-, HMBC-, NOESY- and ROESY-spectra were recorded. The latter two, as well as a ^{1}H-

spectrum with long relaxation time were measured on a cryometer head. All measurements, except ^{19}F-NMR and ^{13}C apt-NMR spectroscopy were performed on the AV 600 instrument. All ^{13}C-NMR measurements were performed proton decoupled. The signal of the respective deuterated solvent (DMSO, $CDCl_3$, CD_3CN) was used as a reference for the internal shift. For the ^{19}F-NMR measurements, TMS was used as a signal reference. Abbreviations used are: s = singlet, d = doublet, t = triplet, q = quartet, m = multiplet, br. s = broad singlet. Coupling constants (J) are expressed in Hz. NMR data were analyzed with MestReNova software. Mass spectra were obtained with two different spectrometers using the same column. LCMS (method 1): instrument: Agilent Technologies 6220 Accurate Mass TOF LC/MS linked to Agilent Technologies HPLC 1200 Series; column: Thermo Accuore RP-MS; particle size: 2.6 µM dimension: 30 × 2.1 mm; Eluent A: H_2O with 0.1% formic acid (FA) Eluent B: MeCN with 0.1% FA; gradient: 0.00 min 95% A, 0.2 min 95% A, 1.1 min 1% A, 2.5 min Stoptime, 1.3 min Posttime; flow rate: 0.8 ml min−1; UV-detection: 220 nm, 254 nm, 300 nm. LCMS (method 2): instrument: Agilent Technologies 6120 Quadrupole LC/MS linked to Agilent HPLC 1290 Infinity; column: Thermo Accuore RP-MS; particle size: 2.6 µM dimension: 30 × 2.1 mm; Eluent A: H_2O with 0.1% FA Eluent B: MeCN with 0.1% FA; gradient: 0.00 min 95% A, 0.2 min 95% A, 1.1 min 1% A, 2.5 min Stoptime, 1.3 min Posttime; flow rate: 0.8 ml/min; UV-detection: 220 nm, 254 nm, 300 nm. Percolated aluminum sheets (Merck Silica gel/TLC-cards, 254 nm) were used for TLC. Purification of the compounds was either performed by flash chromatography using an Isolera System from Biotage or by reversed HPLC using a C18 column on a Prep 150 LC system from Waters.

(Z)-2-Cyano-3-hydroxy-N-(4-(trifluoromethoxy)phenyl)but-2-enamide CF_3O-TF

5-Methyl-N-(4-(trifluoromethoxy)phenyl)isoxazole-4-carboxamide

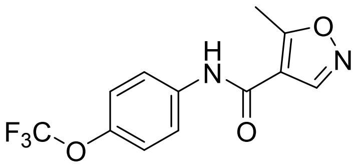

4-(Trifluoromethoxy)aniline (133 mg, 0.75 mmol) was dissolved with $NaHCO_3$ (47.1 mg, 0.55 mmol) in distilled water (0.4 mL) and heated to 50°C. 5-Methyl-4-isoxazolic acid chloride (383 mg, 2.6 mmol) was added slowly, and a colourless solid precipitated immediately. The solution was decanted, the residue was washed with

distilled water (2 x 8 mL) and then dried in high vacuum to yield the product as a colorless amorphous solid (197 mg, 92%). ^{1}H-NMR (600 MHz, Chloroform-*d*) δ = 8.46 (s, 1H, H3), 7.59 (d, $^{3}J_{HH}$ = 8.6 Hz, 2H, H11; H14), 7.47 (s, 1H, H8) 7.22 (d, $^{3}J_{HH}$ = 8.5 Hz, 2H, H10, H14), 2.77 (s, 3H, H16). ^{13}C-NMR (151 MHz, Chloroform-*d*) δ = 173.8 (s, C1), 159.2 (s, C6), 147.4 (s, C3), 145.9 (s, C12), 135.7 (s, C9), 121.9 (s, 2C, C10; C14), 121.7 (s, 2C, C11; C13), 120.4 (q, $^{1}J_{CF}$ = 257.1 Hz, 1C, C17), 111.7(s, C2) , 12.6 (s, C16). ^{19}F-NMR (282 MHz, Chloroform-*d*) δ = 19.4. LCMS (pos. ESI) *m/z:* $[M+H]^+$ (calcd) for $C_{12}H_{10}F_3N_2O_3$ = 287.0638; found: 287.0. HRMS (ESI) m/z: M+ calcd for $C_{12}H_9F_3N_2O_3$ = 286.0565, found: 286.0575.

(Z)-2-Cyano-3-hydroxy-N-(4-(trifluoromethoxy)phenyl)but-2-enamide CF_3O-TF

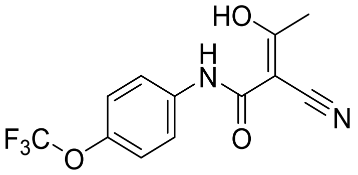

5-Methyl-N-(4-(trifluoromethoxy)-phenyl)isoxazole-4-carboxamide (41.1 mg, 0.14 mmol) was dissolved in ethyl acetate (0.12 mL), mixed with 0.07 mL aqueous NaOH (8.0 mg, 0.20 mmol,) and methanol (0.08 mL) and heated to 55°C for 1h. After cooling to 30°C, distilled water (0.2 mL) and subsequently 2N HCl solution (0.11 mL) was added and stirred for 1 h. The suspension was brought to room temperature, the solvent was decanted, the pink solid was washed three times with distilled water (9 mL) and dried in high vacuum to yield an amorphous solid (24.3 mg, 59%).^{1}H-NMR (600 MHz, DMSO-d_6) δ = 10.68 (s, 1H, H8), 9.92 (s, 1H, H8 (*I*)), 7.69 (d, $^{3}J_{HH}$ = 8.7 Hz, 2H, H11; H16 (*I*)), 7.64 (d, $^{3}J_{HH}$ = 9.0 Hz, 2H, H11; H16), 7.31 (d, $^{3}J_{HH}$ = 8.7 Hz, 2H, H12; H15), 2.36 (s, 3H, H8 (*I*)), 2.25 (s, 3H, H8). ^{13}C-NMR (151 MHz, DMSO-d_6) δ = 187.9 (s, C6), 167.1 (s, C2), 144.6 (s, C13), 137.5 (s, C10), 123.2 (s, 2C, C12; C15), 121.9 (s, 2C, C11; C16), 120.6 (q, $^{1}J_{FF}$ = 255.7 Hz, 1C, C17), 80.5 (s, C3), 23.7 (s, C8). ^{19}F-NMR (282 MHz, DMSO-d_6) δ = 20.5. LCMS (pos. ESI) *m/z:* $[M+H]^+$ (calcd) for $C_{12}H_{10}F_3N_2O_3$ = 287.0638; found: 287.0. HRMS (ESI) m/z: $[M - H]^-$ calcd for $C_{12}H_9F_3N_2O_3$ = 286.0565, found: 286.0575.

(Z)-N-(3,5-Bis(trifluoromethyl)phenyl)-2-cyano-3-hydroxybut-2-enamide di-CF$_3$-TF

N-(3,5-Bis(trifluoromethyl)phenyl)-5-methylisoxazole-4-carboxamide

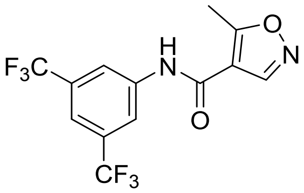

3,5-Bis(trifluoromethyl)aniline (217.6 mg, 0.95 mmol) was suspended with $NaHCO_3$ (62 mg, 0.74 mmol) in distilled water (0.4 mL) and heated to 50°C. 5-Methyl-4-isoxazolic acid chloride (158.4 mg, 1.09 mmol) was added over 5 minutes, resulting in precipitation of a colourless solid. After 20 min the reaction was brought to room temperature, the solvent was decanted and the residue was washed with 1N HCl solution (2 x 10 mL) and distilled water (10 mL). The product was dried in a high vacuum and isolated as colorless amorphous solid (305 mg, 95%). ^{1}H-NMR (600 MHz, DMSO-d_6) δ = 10.58 (s, 1H, H8), 9.06 (s, 1H, H3), 8.39 (s, 2H, H10; H14), 7.83 (s, 1H, H12), 2.71 (s, 3H, H15). ^{13}C-NMR (151 MHz, DMSO-d_6) δ = 174.3 (s, C1), 160.3 (s, C6), 149.3 (s, C3), 141.0 (s, C9), 131.2 (q, $^2J_{CF}$ = 32.8 Hz, 2C, C11; C13), 123.7 (q, $^1J_{CF}$ = 272.7 Hz, 2C, C16; C20), 120.2 (s, 2C, C10; C14), 117.1 (s, C12), 111.9 (s, C2), 12.7 (s, C15). ^{19}F -NMR (282 MHz, Chloroform-*d*) δ = 14.5. LCMS (pos. ESI) *m/z:* $[M+H]^+$ (calcd) for $C_{13}H_9F_6N_2O_2$ = 339.0563; found: 339.1. HRMS (ESI) m/z: M+calcd for $C_{13}H_9F_6N_2O_2$ = 339.0568, found: 339.0535.

(Z)-N-(3,5-Bis(trifluoromethyl)phenyl)-2-cyano-3-hydroxybut-2-enamide di-CF$_3$-TF

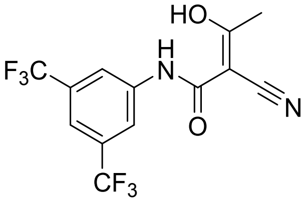

N-(3,5-Bis(trifluoromethyl)phenyl)- 5-methylisoxazole-4-carboxamide (68.4 mg, 0.2 mmol) was placed in ethyl acetate (0.23 mL), mixed with 0.01 mL aqueous NaOH (11.3 mg, 0.28 mmol) and methanol (0.11 mL) and heated to 55°C for 1h. Then distilled water (0.34 mL) and 2N HCl solution (0.2 mL) was added at 30°C and the mixture was stirred for 1 h at 25°C. The solvent was decanted and the product was washed with distilled water (3 x 9 mL) and dried in a high vacuum to yield a slightly

pink amorphous solid (41 mg, 60%). ^{1}H-NMR (600 MHz, DMSO-d_6) δ = 11.54 (s, 1H, H9), 10.29 (s, 1H, H9 (*I*)), 8.34 (s, 2H, H11, H15 (*I*)), 8.25 (s, 2H, H11, H15), 7.75 (s, 1H, H13 (*I*)), 7.68 (s, 1H, H13), 2.42 (s, 1H, H8 (*I*)), 2.21 (s, 3H, H8). ^{13}C-NMR (151 MHz, DMSO-d_6) δ = 188.1 (s, C6), 167.1 (s, C2), 141.3 (s, C10), 131.0 (q, $^{2}J_{FF}$ = 32.6 Hz, 2C, C12; C14), 123.7 (q, $^{2}J_{FF}$ = 272.8 Hz, 2C, C16; C20), 120.5 (s, C4), 120.4 (s, 2C, C11; C15), 115.9 (s, C13), 80.1 (s, C13), 24.9 (s, C8). ^{19}F NMR (282 MHz, DMSO-d_6) δ = 15.9 (s, 6F, F17-19; F21-23), 15.9 (s, 6F, F17-19; F21-23(*I*)). LCMS (pos. ESI) *m/z:* $[M+H]^+$ (calcd) for $C_{13}H_9F_6N_2O_2$ = 339.0563; found: 339.0. HRMS (ESI) m/z: $[M - H]^-$ calcd for $C_{13}H_8F_6N_2O_2$ = 338.049, found: 338.0502.

Z)-2-Cyano-3-hydroxy-N-(4-(pentafluoro-λ⁶-sulfanyl)phenyl)but-2-enamide SF_5-TF

5-Methyl-N-(4-(pentafluoro-λ⁶-sulfanyl)phenyl)isoxazole-4-carboxamide

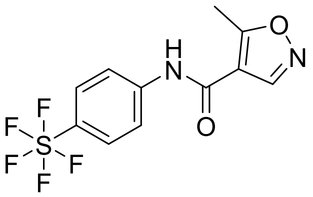

4-(Pentafluorothio)aniline (142.6 mg, 0.65 mmol) was dissolved with $NaHCO_3$ (61.9 mg, 0.74 mmol) in distilled water (0.4 mL) with toluene (0.05 mL) and heated to 50°C. 5-Methyl-4-isoxazolic acid chloride (114.4 mg, 0.78 mmol) was added over 5 minutes, resulting in a colorless solid. After 20 min, the reaction was brought to room temperature, the solvent was decanted and the residue treated with 1N HCl solution (2 x 10 mL) and washed with distilled water (10 mL). The product was dried in a high vacuum and was isolated as a colorless solid (201 mg, 79%). ^{1}H-NMR (600 MHz, DMSO-d_6) δ = 10.41 (s, 1H, H8), 9.09 (s, 1H, H3), 7.94 – 7.88 (m, 4H, H10; H14; H11; H13), 2.69 (s, 3H, H21). ^{13}C-NMR (151 MHz, DMSO-d_6) δ = 174.0 (s, C1), 160.1 (s, C6), 149.5 (s, C3), 148.4 – 147.8 (m, 1C, C12), 142.4 (s, C9), 127.2 (s, 2C, C11; C13), 120.2 (s, 2C, C10, C14), 112.1 (s, C2), 12.7 (s, C21). ^{19}F-NMR (282 MHz, DMSO-d_6) δ = 166.1 (p, $^{2}J_{FF}$ = 149.3 Hz, 1F, F19), 142.4 (d, $^{2}J_{FF}$ = 151.0 Hz, 4F, F16; F17; F18; F20). LCMS (pos. ESI) *m/z:* $[M+H]^+$ (calcd) for $C_{11}H_{10}F_5N_2O_2S$ = 329.0378; found: 329.0. HRMS (ESI) m/z: M+ calcd for $C_{11}H_{10}F_5N_2O_2S$ = 329.0383, found: 329.0347.

(Z)-2-Cyano-3-hydroxy-N-(4-(pentafluoro-λ[6]-sulfanyl)phenyl)but-2-enamide SF_5-TF

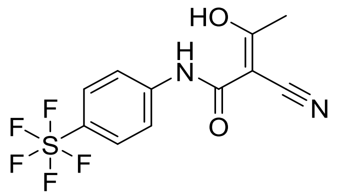

5-Methyl-N-(4-(pentafluorothio)phenyl)isoxazole-4-carboxamide (87.4 mg, 0.27 mmol) was dissolved in ethyl acetate (0.23 mL), mixed with 0.13 mL aqueous NaOH (15.20 mg, 0.38 mmol) and methanol (0.15 mL) and heated to 55°C for 1h. Then distilled water (0.39 mL) and 2N HCl solution (0.21 mL) was added at 30°C and the mixture stirred for 1 h at 30°C. The solvent was decanted, the product was washed three times with distilled water (9 mL) and dried in a high vacuum to yield a slightly pink amorphous solid (60.0 mg, 68%). ^{1}H-NMR (600 MHz, DMSO-d_6) δ = 11.19 (s, 1H, H9), 10.16 (s, 1H, H9 (I)), 7.81 (d, J = 9.3 Hz, 2H, H12; H15), 7.75 (d, J = 9.1 Hz, 2H, H11; H16), 2.38 (s, 1H, H8 (I)), 2.21 (s, 3H, H8). ^{13}C-NMR (151 MHz, DMSO-d_6) δ = 187.7 (s, C6), 166.8 (s, C2), 149.3 – 146.1 (m, 1C, C13), 142.4 (s, C10), 127.0 (s, 2C, C12; C15), 120.4 (s, 2C, C11; C16), 120.2 (s, C4), 80.6 (s, C3), 24.5 (s, C8). ^{19}FNMR (282 MHz, DMSO-d_6) δ = 166.4 (p, $^{2}J_{FF}$ = 152.5 Hz, 1F, F19), 142.5 (d, $^{2}J_{FF}$ = 151.0 Hz, 4F, F17; F18; F20; F21). LCMS (pos. ESI) m/z: [M+H]+ (calcd) for $C_{11}H_{10}F_5N_2O_2S$ = 329.0378; found: 329.1. HRMS (ESI) m/z: [M - H]- calcd for $C_{11}H_9F_5N_2O_2S$ = 328.0305, found: 328.0311.

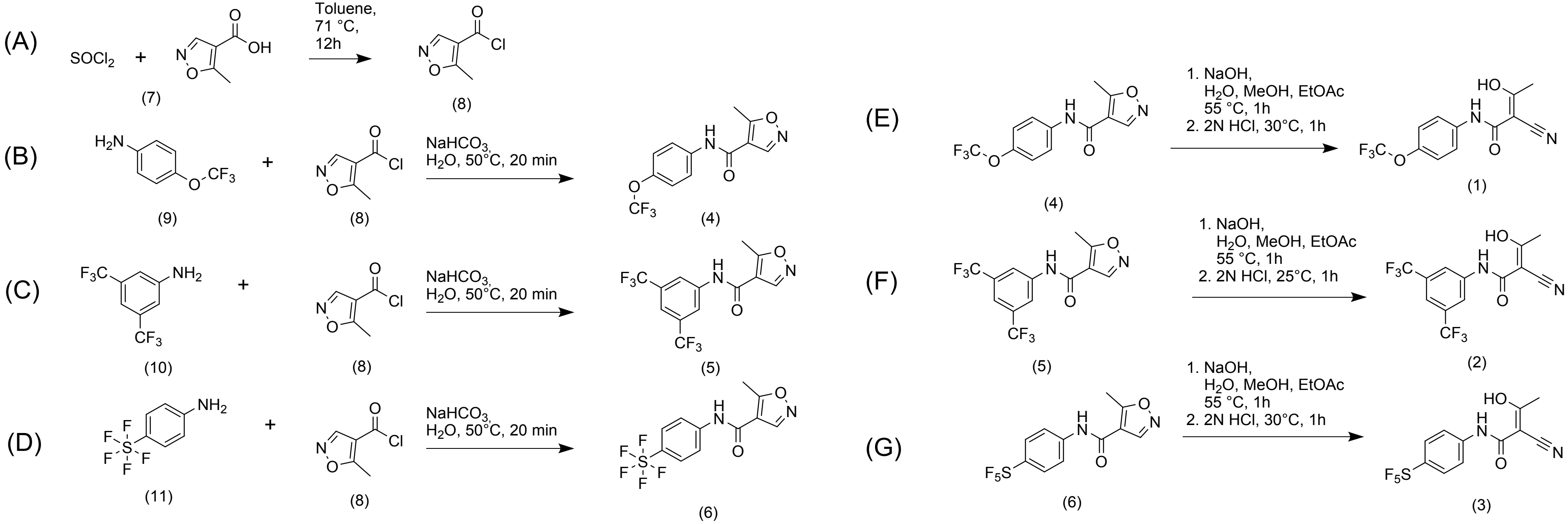

Figure S1. Synthesis of leflunomide (B-D) and teriflunomide (E-G) derivatives. A. Synthesis of isoxazole acid chloride, B. Synthesis of CF_3O-LF (4), C. Synthesis of *di*-CF_3-LF (5), D. Synthesis of SF_5-LF (6), E. Synthesis of CF_3O-TF (1), F. Synthesis of *di*-CF_3-TF (2), G. Synthesis of SF_5-TF (3).

Flow cytometry

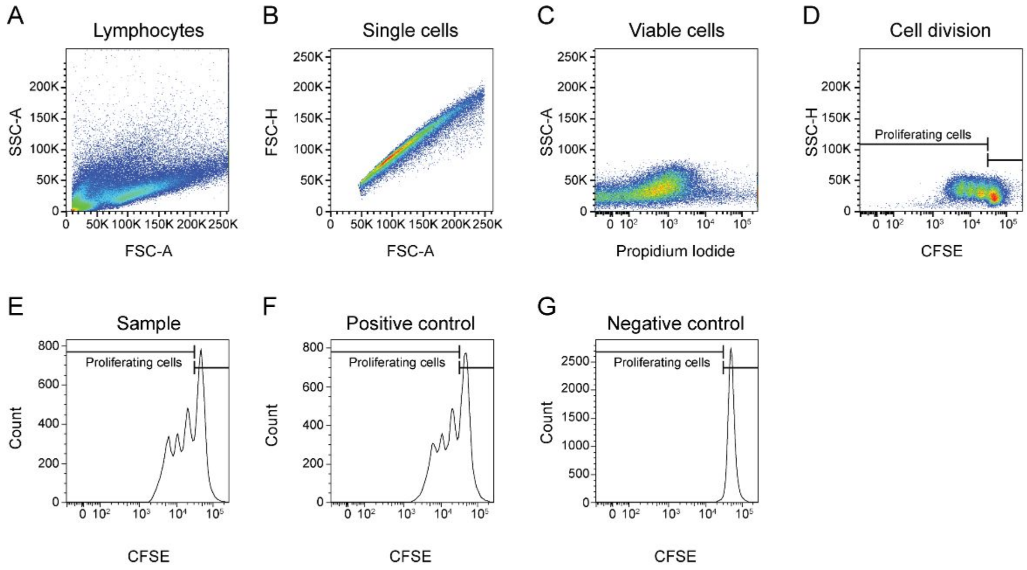

Figure S2. Flow cytometry analysis of proliferating cells. A. Selection of the lymphocyte population, **B.** Selection of single cells only, **C.** Selection of propidium iodide (PI) negative cells, while excluding death, thus stained cells, **D.** Gating for CFSE positive cells, showing 4 different subpopulations of cells with different proportions of CFSE positive cells; the bar divides non-proliferating cells from proliferating cells, **E.** Histogram of CFSE positive cells (treated with teriflunomide) and differentiation and quantification of non-proliferating (right hand side) from proliferating cells (left from the non-proliferating peak), **F.** Histogram of CFSE positive cells (without treatment but with proliferation stimulus; positive control), **G.** Histogram of CFSE positive cells (without treatment and without stimulus; negative control).

Enzyme inhibition and cell growth inhibition

Dose response model, including outlier detection and IC_{50} calculation for DHODH and CFSE data

The analysis of raw data from DHODH inhibition assays and CFSE cell proliferation assays was performed in R (version 3.6.1, R Foundation) using the drc R package for fitting a four-parameter logistic dose-response model to the data points [1, 2]:

$$f(x(b,c,d,e)) = c + \frac{d-c}{1+\exp(b(log(x)-log(e)))},$$

[b: steepness = Hill slope, c: upper limit, d: lower limit, e: halfway through point = point of inflection].

Outliers were detected and excluded from an initially robust fitting according to Motulsky and Brown [3] followed by a refitting of the data.

From the model, we calculated the drug concentration inhibiting 50 % (IC_{50}) of either the enzymatic activity in DHODH inhibition assays or the proliferation of stimulated cells in CFSE cell proliferation assays for each compound (Figure S3). A 95 % confidence interval was calculated for each fit, indicating the concentration range which includes the IC_{50} with a probability of 95 %.

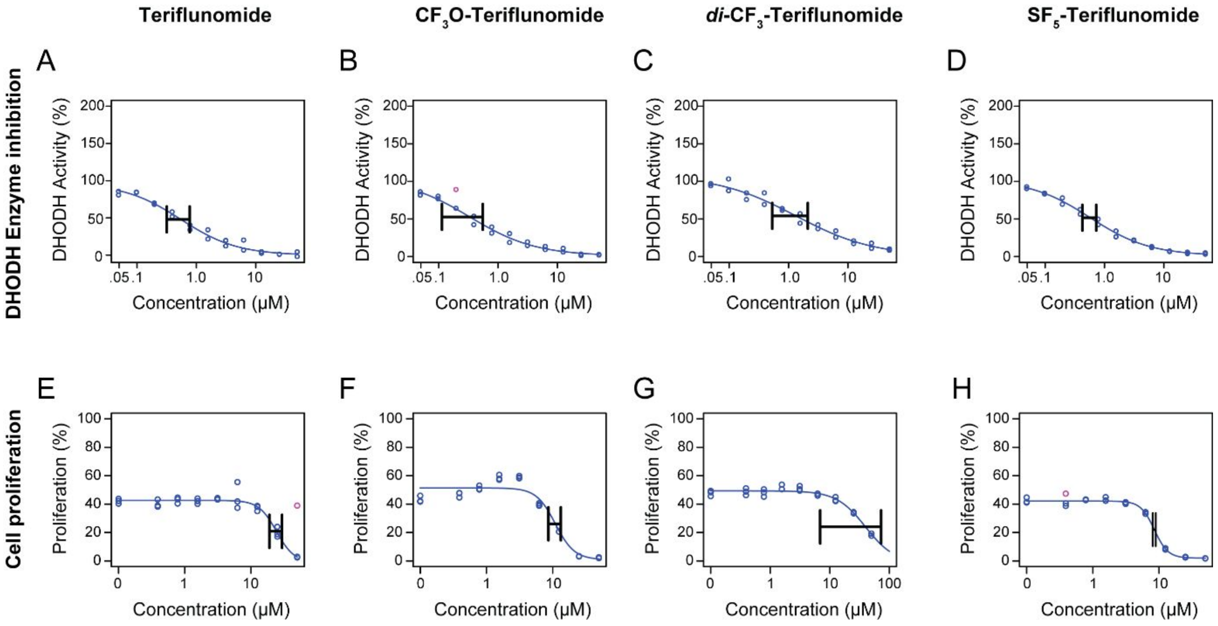

Figure S3. Biological activity of teriflunomide (TF) derivatives. Dose response curves from DHODH (A-D) and cell proliferation assays (E-H) to assess the biological activity of teriflunomide derivatives. The reduction in activity in DHODH activity (duplicate measurements) and the proportion of proliferating cells in cell proliferation assays is depicted vs. increasing concentration of inhibitor. A dose-response model was fitted to the data points. Outliers were detected and excluded from the fitting (magenta). A 95% confidence interval is depicted in the plots.

Toxicology

Plate design and dilutions

For assessing the cytotoxicity of compounds, we performed a cytotoxicity test in HepG2 cells. Experiments were performed in 384 well plates (cellbind plate, Corning) in triplicates at seven concentrations ranging from 1.5 µM to 100 µM (DMSO concentration was 0.53 % in all wells). 5-FU (1 mM) was used as positive toxicity control and DMSO alone as negative control.

Cell culture

HepG2 cells were cultured in RPMI medium (10 % FCS) with 1800 cells per well. Compounds were added on the following day, and plates were incubated for 72 h.

Assay

Following the incubation period, cells were fixed, stained, and measured using an automated widefield fluorescence microscope (Arrayscan XTI/Thermo). Hoechst 3342 (Sigma Aldrich, final concentration 1 µM, nuclear staining) and Yo-Pro-1 (Invitrogen, final concentration 0.1 µM, apoptosis assay) was used for live cell staining (1 h incubation). The live cell measurement microscopy was performed on two fluorescence channels at 20x magnification. The number of non-proliferating (Hoechst 3342^{+}) and dead (Yo-Pro-1^{+}) cells were quantified. Following fixation, viable cells were counted by microscopy, using 10x magnification.

Comparison of growth inhibition and toxicology

We performed a growth inhibition assay and a cytotoxicity screening of TF and its derivatives in HepG2 cells to study their impact on liver cells as drug entrance and metabolic compartment. All compounds showed inhibitory activity in the concentration range of 1.5 µM to 100 µM. Cytotoxicity was assessed by quantifying damaged cells by apoptosis markers. TF did not show any cytotoxic effect in this concentration range compared to untreated negative controls. Equally, CF_3O-TF did not display cytotoxic effects up to 100 µM. *di*-CF_3-TF showed no cytotoxicity up to 50 µM of concentration. SF_5-TF showed minimal cytotoxic effects.

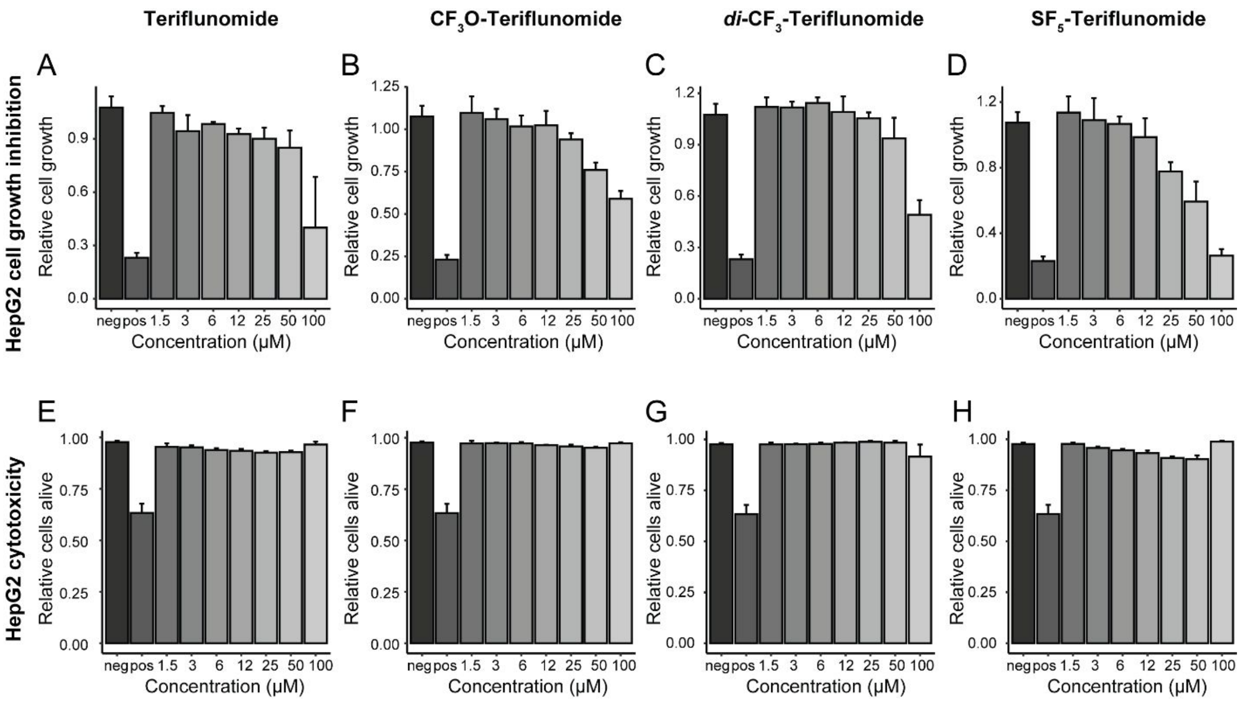

Figure S4. Inhibitory activity and cytotoxicity of teriflunomide compounds in HepG2 cells. A-D. Cell growth inhibition of HepG2 cells at concentrations ranging from 1.5 to 100 µM, **E-H.** Surviving cells indicating cytotoxicity in HepG2 cells at concentrations ranging from 1.5 to 100 µM.

Additionally, we determined the cytotoxic effect of TF derivatives on PBMCs using a propidium iodide (PI) staining. In two patients and in comparison to untreated cells (both unstimulated and stimulated cells), the amount of surviving cells was above 90 %.

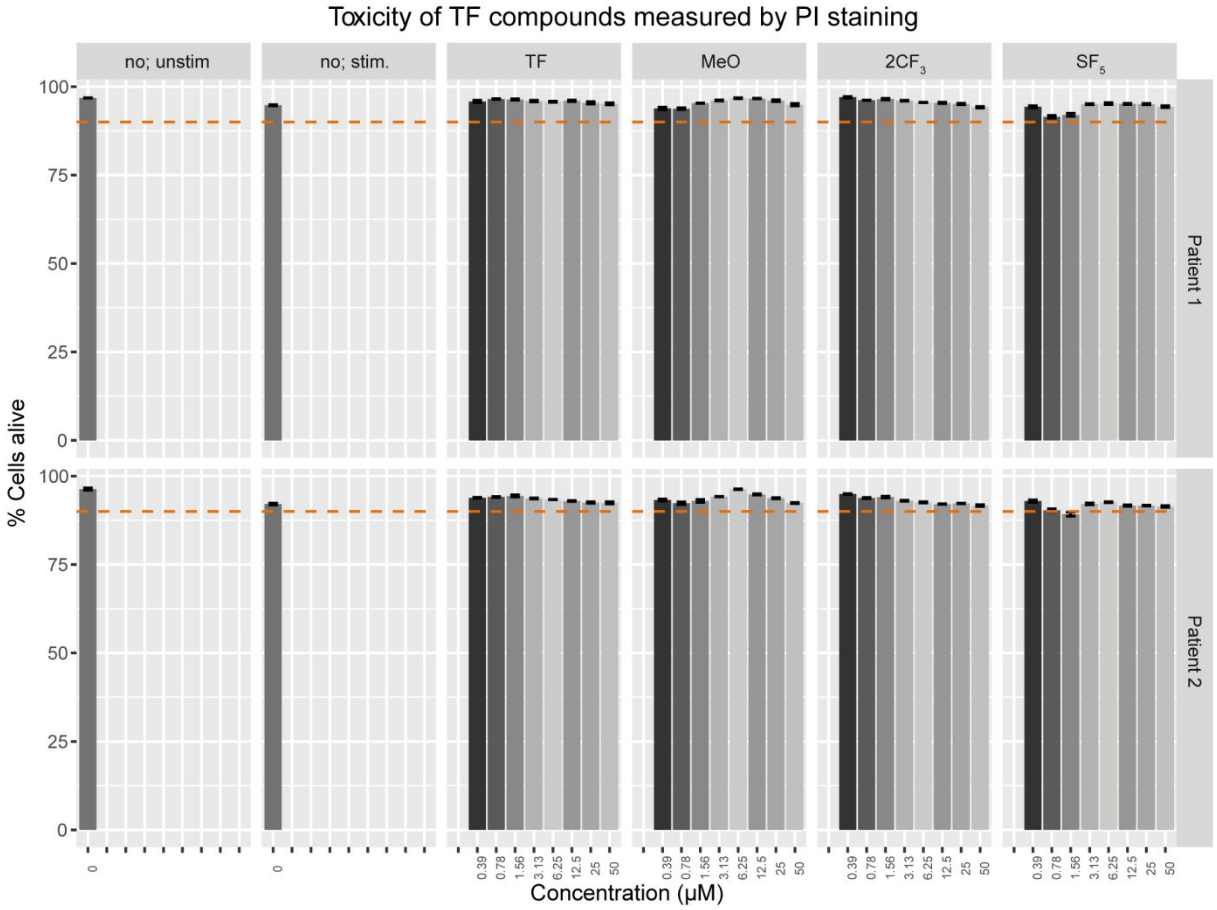

Figure S5. Cytotoxicity of teriflunomide compounds in PBMCs. Surviving cells in PBMCs at concentrations ranging from 0.39 to 50 μM, determined by propidium iodide (PI) staining after 72 h of incubation of cells with TF compounds. The experiment was performed in 2 individual patients, the orange line indicates the extent of at least 90 % of cell survival.

MR characterization

Table S1. Concentrations of TF compounds in DMSO and serum phantoms

Compound	DMSO (mg/mL)	Serum (mg/mL)
**TF**	21.48	4.84
**CF_3O-TF**	20.28	2.00
***di*-CF_3-TF**	8.58	6.98
**SF_5-TF**	10.67	3.47

Table S2. Full width half maximum (FWHM) of TF compounds in DMSO and serum

Compound	DMSO (ppm)	Serum (ppm)
**TF**	0.65	1.42
**CF_3O-TF**	0.71	1.35
***di*-CF_3-TF**	0.72	1.26
**SF_5-TF**	1.41	3.29

Table S3. Optimized sequence parameters for SNR efficiency measurements

Compound	TF		CF_3O-TF		*di*-CF_3-TF		SF_5-TF	
**Medium (RT)**	DMSO	Serum	DMSO	Serum	DMSO	Serum	DMSO	Serum
**TR for RARE (ms)**	1968	1305	3610	1026	1943	1130	596	389
**ETL for RARE**	48	8	96	16	48	8	8	4
**bSSFP flip angle (°)**	71	8	74	12	75	9	46	15
**Ernst angle (°)**	25	25	20	19	24	27	40	42

^{19}F MR Images of the DMSO and serum phantoms

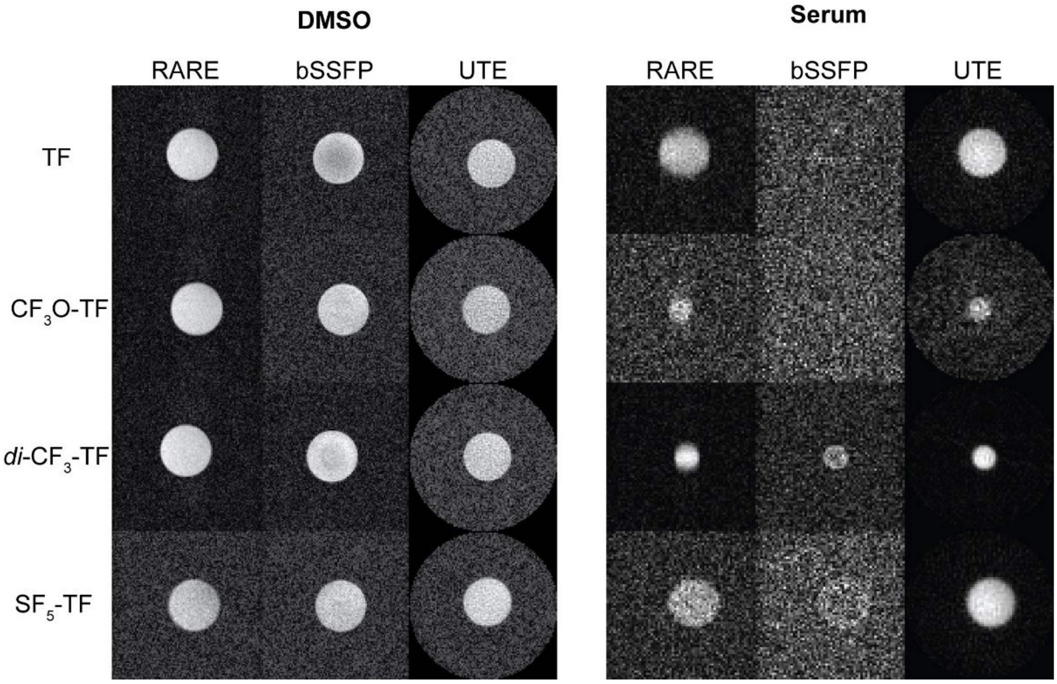

Figure S6. ^{19}F MR Images of phantoms containing TF compounds dissolved in DMSO and in serum. Optimized scan parameters were used and the SNR efficiency normalized to the concentrations in the phantoms was compared.

^{19}F MR spectral line splitting of SF_5-TF in DMSO

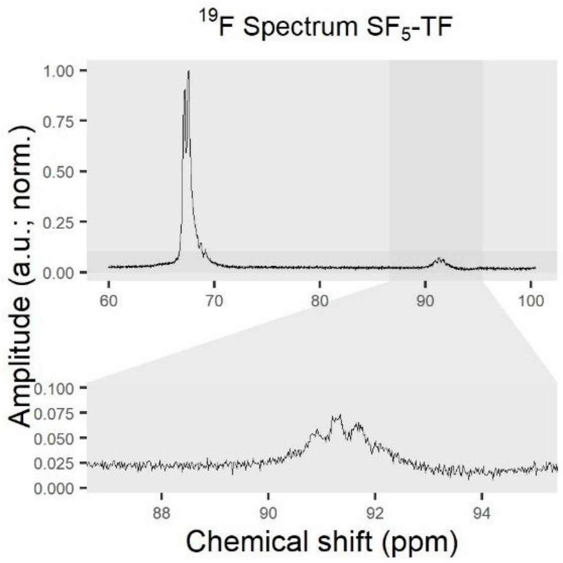

Figure S7. ^{19}F MR spectroscopy of a phantom containing TF dissolved in DMSO. The doublet at 67.4 ppm is shown separately from the pentet at 91.7 ppm.

Biological and ^{19}F MR reporter activity of TF and SF_5-TF

We administered TF and SF_5-TF orally to C57BL/6 mice (n = 6) using the same molar concentrations: TF =12.15 mg/ml (n = 3) and SF_5-TF = 10 mg/ml (n = 3). ^{19}F MR UTE MR images were then acquired using optimized parameters. Peak ^{19}F SNR values for each compound were calculated for all 6 mouse stomachs (**Table S4**). These values were calculated from the ^{19}F MR images of TF and SF_5-TF (**Figure S8**).

Table S4. Peak SNR values for ^{19}F MR UTE MR images of TF and SF_5-TF

Mouse	Compound	Figure	Peak SNR
**1**	TF	S8A	6.8492
**2**	TF	S8B	8.0581
**3**	TF	S8C	2.5269
**4**	SF_5-TF	S8D	14.4034
**5**	SF_5-TF	S8E	12.0337
**6**	SF_5-TF	S8F	13.197

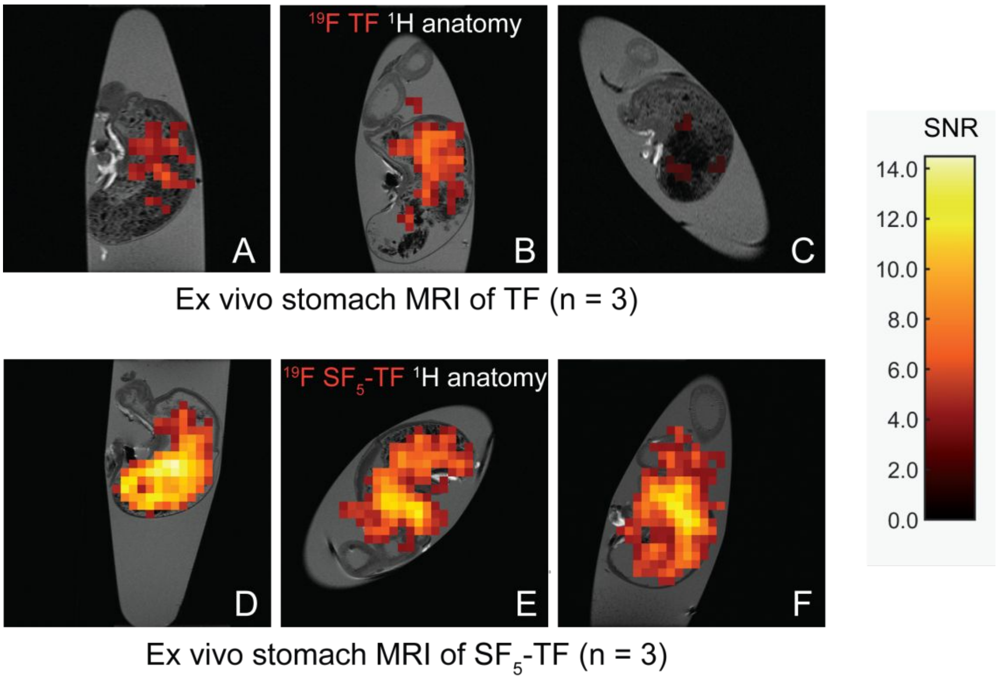

Figure S8. ^{19}F MR reporter activity of TF (A-C) and SF_5-TF (D-F) in the stomach of C57BL/6 mice ex vivo. All ^{1}H RARE anatomical images: TR = 2 s, TE = 10 ms, TA = 1 min, 4 s. ^{19}F UTE MRI of TF: TR = 100 ms, TE = 0.27 ms, FA = 25 °. ^{19}F UTE MRI of SF_5-TF: TR = 100 ms, TE = 0.27 ms, FA = 42 °. All acquisition times (TA) for ^{19}F UTE MRI were 2 h, 30 min, except for the third TF-treated stomach (C). Due to the low signal-to-noise ratio (SNR) for this sample (C) we used a TA = 12 h, 30 min (5x longer acquisition time) and SNR was then downscaled by $\sqrt{5}$ and this value indicated in Table S4. SNR is indicated by the color bars.

References

1. Ritz, C.; Baty, F.; Streibig, J. C.; Gerhard, D., Dose-Response Analysis Using R. *PLoS One* **2015,** *10* (12), e0146021.

2. Gadagkar, S. R.; Call, G. B., Computational tools for fitting the Hill equation to dose-response curves. *J Pharmacol Toxicol Methods* **2015,** *71*, 68-76.

3. Motulsky, H. J.; Brown, R. E., Detecting outliers when fitting data with nonlinear regression - a new method based on robust nonlinear regression and the false discovery rate. *BMC bioinformatics* **2006,** *7*, 123.
